# Synergistic interaction between sorafenib and gemcitabine in EGFR-TKI-sensitive and EGFR-TKI-resistant human lung cancer cell lines

**DOI:** 10.3892/ol.2012.1017

**Published:** 2012-11-07

**Authors:** JING LI, YUE-YIN PAN, YING ZHANG

**Affiliations:** 1 Department of Geriatrics, The Third Affiliated Hospital of Anhui Medical University, Hefei 230061;; 2 Department of Oncology, The First Affiliated Hospital of Anhui Medical University, Hefei 230022, P.R. China

**Keywords:** sorafenib, gemcitabine, non-small cell lung cancer, sequence treatment

## Abstract

Sorafenib is a highly selective multi-targeted agent and has been reported to have potent antitumor effects against various tumors, including human non-small cell lung cancer (NSCLC). In the present study, we explored the antitumor effect and associated molecular mechanisms of sorafenib against human lung cancer cell lines *in vitro*. We also investigated the efficacy of concurrent and sequential administration of sorafenib and gemcitabine in epidermal growth factor receptor (EGFR)-tyrosine kinase inhibitor (TKI)-sensitive and EGFR-TKI-resistant NSCLC cell lines. The PC-9 (EGFR-TKI-sensitive, EGFR-mutated) and A549 (EGFR-TKI-resistant, K-Ras-mutated) NSCLC cell lines were treated with sorafenib and gemcitabine, alone, in combination or with different schedules. Cytotoxicity was assessed by MTT assay, cell cycle distribution was analyzed by flow cytometry and alterations in signaling pathways were analyzed by western blotting. We found that sorafenib exhibited dose-dependent growth inhibition in the EGFR-TKI-sensitive and EGFR-TKI-resistant NSCLC cell lines, and the sequence gemcitabine→sorafenib exhibited the strongest synergism. Sorafenib arrested the cell cycle at G1 phase, whereas gemcitabine caused arrest at S phase. The molecular mechanism of this synergism is that the downstream signaling pathways that were initially activated by gemcitabine exposure were efficiently suppressed by the subsequent exposure to sorafenib. By contrast, the reverse of this sequential administration resulted in antagonism, which may be due to differential effects on cell cycle arrest. The results suggest that sorafenib as a single agent exhibits anti-proliferative effects *in vitro* in NSCLC cell lines with EGFR and K-Ras mutations and that the sequential administration of gemcitabine followed by sorafenib is superior to sorafenib followed by gemcitabine and concurrent administration.

## Introduction

Lung cancer is a global health issue and the leading cause of cancer-related mortality. Non-small cell lung cancer (NSCLC) accounts for 80–85% of all lung cancer cases (1). Despite the optimization of chemotherapy regimens, treatment outcomes for advanced NSCLC remain disappointing.

Gefitinib and erlotinib are orally administered, small-molecule epidermal growth factor receptor (EGFR)-tyrosine kinase inhibitors (TKIs) that improve the survival of NSCLC patients and caused a paradigm shift for the treatment of NSCLC. Patients with EGFR-activating mutations greatly benefit from treatment with EGFR-TKIs (2–4). However, the presence of K-Ras mutation is associated with primary resistance to EGFR-TKIs (5,6). In NSCLC, 15–30% of adenocarcinoma patients possess a gain of function mutation in the K-Ras gene, meaning that for these patients, their tumors fail to respond to EGFR-TKIs (7,8). Thus, clinical research of new treatment strategies for NSCLC patients is urgently needed.

Angiogenesis is a complex process regulated by several pro- and anti-angiogenic factors. Vascular endothelial growth factor (VEGF) and platelet-derived growth factor (PDGF) are critical factors in the promotion of angiogenesis in NSCLC (9,10). Activation of VEGF and PDGF stimulated downstream signaling pathways, including phosphatidylinositol-3-kinase (PI3K) and extracellular signal-regulated kinase (ERK) (11–13). The Ras/Raf/MEK/ERK and Ras/PI3K/PTEN/Akt pathways interact to regulate growth and play key roles in the transmission of proliferative signals. Therefore, the overexpression of VEGF and PDGF is correlated with tumor progression of NSCLC patients and is a strong prognostic indicator in NSCLC (14–16). In NSCLC, activation of K-Ras leads to ERK1/2 overexpression through the Raf/MEK/ERK signaling pathway (17–19). Hence, inhibition of the Ras/RAF/MEK/ERK signaling pathway is an important strategy in anticancer drug development.

Sorafenib (BAY 43-9006) is an oral multikinase inhibitor that decreases the activity of C-RAF and B-RAF in the RAF/MEK/ERK signaling pathway and targets the VEGF receptor family (VEGFR-2 and VEGFR-3) and PDGF receptor familyβ (PDGFRβ) (20). Single-agent sorafenib showed preclinical and clinical activity against NSCLC (21–23). In xenograft models administered a combination of sorafenib and anticancer agents, such as vinorelbine, cisplatin and gefitinib, the anti-proliferative effect is at least as efficacious as sorafenib alone and the treatment is well-tolerated (24). The safety profile of sorafenib in previous trials has increased the feasibility of using the drug in combination with cytotoxic and cytostatic agents. However, sorafenib administered concurrently with chemotherapy does not improve patient outcomes compared with chemotherapy alone in advanced-stage NSCLC. The ESCAPE trial assessed the efficacy and safety of sorafenib in combination with carboplatin and paclitaxel in 926 patients with advanced NSCLC. There was no clinical benefit observed from adding sorafenib to carboplatin and paclitaxel (CP) chemotherapy as first-line treatment for NSCLC. Patients with squamous cell histology had greater mortality (25). The subsequent NExUS trial of sorafenib in combination with gemcitabine/cisplatin in a planned 900 patients with non-squamous advanced NSCLC (NCT00449033) was also stopped early as it failed to meet its primary endpoint of OS (26).

One potential explanation for this lack of benefit is a negative interaction or antagonism between chemotherapy and sorafenib when delivered concomitantly. Support for this line of reasoning is provided by preclinical data demonstrating that sorafenib induce primarily a cytostatic effect resulting from a G1 cell cycle arrest in NSCLC cell lines (27,28), reducing cell cycle phase-dependent (S and G2/M phase) cytotoxicity of chemotherapy. At present, sequential administration is considered to be a promising therapeutic approach in NSCLC as well as in other types of cancer. Sequential administration avoids potential negative interactions between the two drugs and has been explored with EGFR-TKIs and chemotherapy (29,30).

Gemcitabine is a pyrimidine nucleoside antimetabolite agent with a favorable toxicity profile, which is active against a variety of human malignancies, including NSCLC (31), and has been frequently used in combinatorial treatments with other anticancer agents.

In the present study, we used NSCLC cells harboring EGFR and K-Ras mutations to investigate the effect of sorafenib and gemcitabine as single agents and in different sequences on proliferation and cell cycle progression *in vitro*. We also evaluated the molecular mechanisms of the different effects.

## Materials and methods

### Drugs

Sorafenib (BAY 43-9006) was obtained from Bayer (Leverkusen, Germany) and was dissolved in dimethyl sulfoxide (DMSO) to a stock concentration of 10 mmol/l. Gemcitabine was purchased as a commercial product from the pharmacy at The Third Affiliated Hospital of Anhui Medical University, Hefei, China, and was dissolved in DMSO at 100 mmol/l, as stock solution. The drugs were stored at −20°C and diluted with culture medium prior to use.

### Cell lines

The EGFR-TKI-sensitive PC-9 (mutant EGFR/wild-type K-Ras) and EGFR-TKI-resistant A549 (wild-type EGFR/mutant K-Ras) human NSCLC cell lines were purchased from American Type Culture Collection (ATCC, Manassas, VA, USA) and maintained in RPMI-1640 medium (Hyclone, Logan, UT, USA), supplemented with 10% heat-inactivated fetal bovine serum (Hyclone), penicillin (100 U/ml), streptomycin (100 *μ*g/ml) and L-glutamine (2 mM) at 37°C in a 5% CO_2_ atmosphere, and then harvested with trypsin-EDTA when the cells reached exponential growth.

### Anti-proliferative effects of single agents

The anti-proliferative effects of sorafenib and gemcitabine as single agents on A549 and PC-9 cells were evaluated by MTT assay, as previously described (32). Cells were cultured in 96-well plates, in which the number of A549 and PC-9 cells was 4,000 and 6,000 per well, respectively. The IC50 value, indicating the concentration resulting in inhibition of 50% of the maximal cell growth, was determined following 72 h exposure to the drug compared with unexposed control cells. After cells were exposed to each drug for 72 h in 96-well plates, 20 ml MTT solution was added to each well. The optical density (OD) of each well was measured at 490 nm following incubation for 4 h. The percentage of cell growth inhibition resulting from each drug was calculated as: [(OD 490 control cells – OD 490 treated cells)/OD 490 control cells] × 100. This assay was repeated in more than three independent experiments.

### Anti-proliferative effects of different sequences of sorafenib and gemcitabine

The anti-proliferative effects of three different sequences of sorafenib and gemcitabine were evaluated. In the first schedule, cells were pretreated with gemcitabine for 24 h, followed by a washout with phosphate-buffered saline (PBS) and an additional exposure to sorafenib for 72 h. In the second schedule, the reverse sequence of sorafenib followed by gemcitabine was performed. Thirdly, cells were concurrently treated with sorafenib and gemcitabine for 72 h and incubated in a drug-free medium for 24 h. The combination drug doses using constant ratios of the IC50 values were calculated from the previous cytotoxicity tests. Thus, the combination index (CI) value was calculated using 0.125, 0.25, 0.5, 1, 2 and 4 times (A549) or 0.296, 0.444, 0.667, 1, 1.5 and 2.25 times (PC-9) the IC50 of sorafenib and gemcitabine combination doses. The CI values of interactions between sorafenib and gemcitabine were analyzed according to the Chou and Talaly method using CompuSyn software (ComboSyn, Inc., Paramus, NJ, USA): CI>1, CI=1 and CI<1 indicate antagonistic, additive and synergistic effects, respectively (33).

### Cell cycle analysis of single agents and of different sequences of sorafenib and gemcitabine

Cells (1×10^5^/well) were plated into six-well plates and exposed to sorafenib and gemcitabine as single agents and in different sequences at the concentration of IC50 levels for the interval as described above. At the end of each exposure, cells were collected and fixed with 70% cold ethanol at 4°C overnight. DNA staining was performed using a solution with propidium iodide (0.05 mg/ml) and RNase (2 mg/ml) for 30 min at room temperature. Cells were analyzed using a FACScan cytometer and the percentage of cells in G1, S and G2/M phases of the cell cycle was estimated by Cell Lab Quanta SC Software.

### Western blot analysis

Cells (5×10^5^/well) were treated with sorafenib and gemcitabine as single agents and in different sequences for the desired time. Cells were washed with ice-cold PBS solution and scraped in lysis buffer. The lysates were centrifuged at 14,000 rpm for 30 min at 4°C and the supernatant was collected. Equivalent amounts of protein were analyzed by sodium dodecyl sulfate-polyacrylamide gel electrophoresis (SDS-PAGE) and transferred to PVDF membranes. Appropriate primary antibodies to pPDGFRβ, PDGFRβ, pAKT, AKT, pERK1/2, ERK1/2, Bcl-2 and β-actin purchased from Cell Signaling Technology (Beverly, MA, USA) were used. Proteins were visualized with a horseradish peroxidase-coupled secondary antibody from Cell Signaling Technology. Specific bands were detected using the enhanced chemiluminescence reagent (ECL; PerkinElmer Life Sciences, Inc., Boston, MA, USA) on autoradiographic film and quantitated by densitometry.

### Statistical analysis

The results obtained from at least three independent experiments are expressed as mean ± standard deviation (SD). Student’s t-test and one-way ANOVA test were used to determine the differences between control and treatment groups. P<0.05 was considered to indicate a statistically significant result.

## Results

### Dose-dependent anti-proliferative activity of sorafenib and gemcitabine

**Figure 1.**
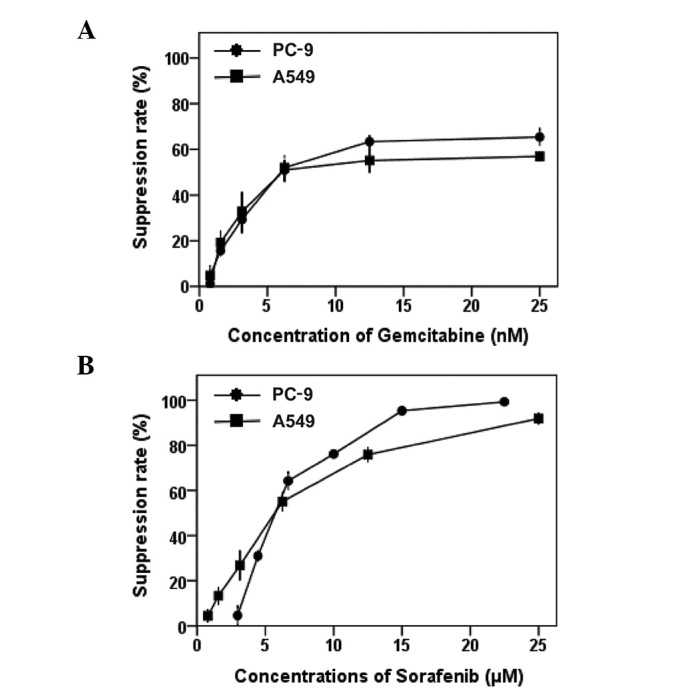
*In vitro* effects of sorafenib and gemcitabine on proliferation of NSCLC cell lines. MTT assays were used to examine the inhibitory activities on cell proliferation. The cells were exposed to the varying concentrations of (A) gemcitabine in A549 cells (0.78–25 nmol/l) and PC-9 cells (0.78–25 nmol/l) or (B) sorafenib in A549 cells (0.78–25 *μ*mol/l) and PC-9 cells (2.96–22.5 *μ*mol/l) for 72 h. Each data point was repeated in more than three independent experiments. NSCLC, non-small cell lung cancer.

MTT assays were used to evaluate the anti-proliferative effects of sorafenib and gemcitabine as single agents on EGFR-TKI-sensitive PC-9 (mutant EGFR/wild-type K-Ras) and EGFR-TKI-resistant A549 (wild-type EGFR/mutant K-Ras) NSCLC cell lines. Dose-dependent growth inhibitory effects of sorafenib (0.78–25 *μ*M) and gemcitabine (0.78–25 nM) were observed in the two NSCLC cell lines (Fig. 1). We demonstrated that the sensitivity of PC-9 and A549 cells to sorafenib or gemcitabine are similar. Table I summarizes the IC50 of the two drugs. The IC50 values of sorafenib in the two cell lines are within the clinically relevant concentration range for this drug (8.5–15.7 *μ*mol/l) (34).

### Schedule-dependent anti-proliferative activity of sorafenib and gemcitabine

**Figure 2.**
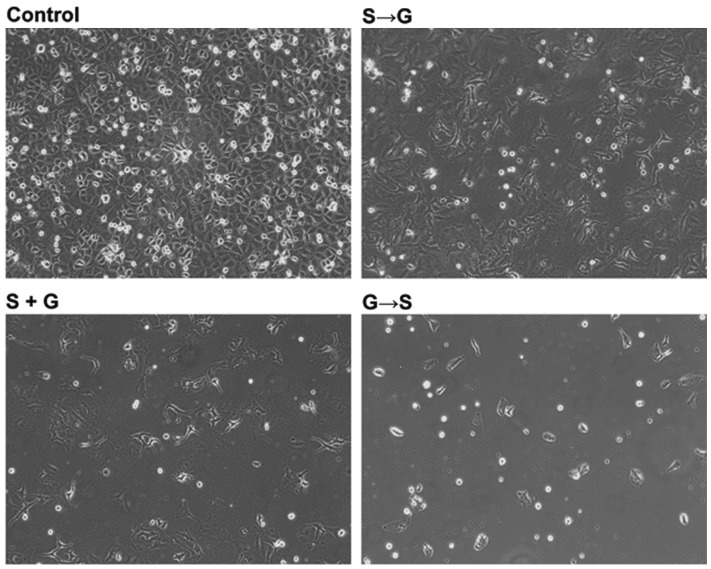
A549 cells were exposed to different schedules at IC50 levels. (OLYMPUS IX70; magnification, ×200). S→G, G→S and S+G refer to sorafenib followed by gemcitabine, gemcitabine followed by sorafenib and concurrent administration, respectively. The anti-proliferative effects of sequential administration of gemcitabine followed by sorafenib were more pronounced than sorafenib followed by docetaxel and concurrent administration of the two drugs. IC50, concentration resulting in inihibition of 50% of maximal cell growth.

**Figure 3.**
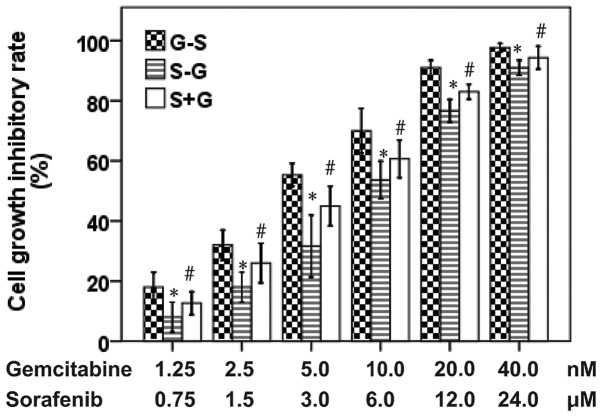
A549 cells were incubated with the constant-ratio dose in three sequences. The concentrations applied for the cells were 1.25–40.0 nM for gemcitabine and 0.75–24.0 *μ*M for sunitinib. G→S, S→G and S+G refer to gemcitabine followed by sorafenib, sorafenib followed by gemcitabine and concurrent administration, respectively. ^*^G→S relative to S→G, P<0.05; ^#^G→S relative to S+G, P<0.05.

**Figure 4.**
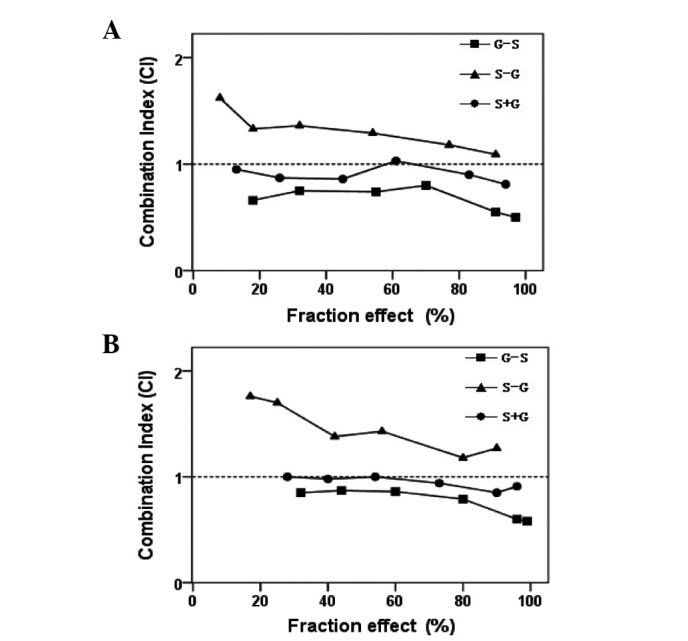
Combination index (CI) value of each drug fraction was calculated using the Chou-Talalay method as described in Materials and methods, in (A) A549 and (B) PC-9 cells following exposure to the different sequences. G→S, S→G, and S+G refer to gemcitabine followed by sorafenib, sorafenib followed by gemcitabine and concurrent administration, respectively. In the two cell lines, CI<1 was detected at every drug concentration with the sequence of gemcitabine followed by sorafenib.

We evaluated the anti-proliferative effects of sorafenib and gemcitabine in three different sequences on A549 and PC-9 cell lines. As shown in Figs. 2 and 3, the anti-proliferative effects observed in A549 cells following the administration of gemcitabine followed by sorafenib were more noticeable than the reversed sequence of sorafenib followed by gemcitabine (P<0.05) and the concurrent administration of the two drugs (P<0.05). Similar results were also found in PC-9 cells. In the A549 and PC-9 cell lines, the calculation of CI values revealed that the sequence of gemcitabine followed by sorafenib produced synergistic effects (Fig. 4), with mean CI values of 0.67 in A549 and 0.76 in PC-9 cells. Concomitant administration of the drugs resulted in synergistic effects, with mean CI values of 0.9 in A549 and 0.95 in PC-9 cells. The sorafenib followed by gemcitabine sequence resulted in an antagonistic interaction with mean CI values of 1.31 in A549 and 1.45 in PC-9 cells. Therefore, regardless of the mutation status of EGFR or K-Ras in NSCLC cells, exposure to gemcitabine followed by sorafenib was shown to exert synergistic effects, whereas the effect of the reversed sequence is antagonistic. These results illustrate that the sequential administration of gemcitabine followed by sorafenib is superior to sorafenib followed by gemcitabine and concurrent administration.

### Cell cycle effects of sorafenib and gemcitabine

**Figure 5.**
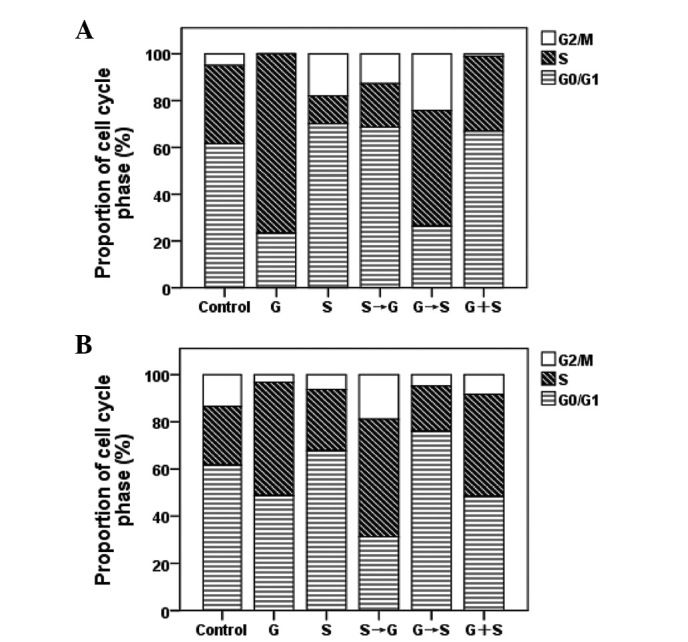
Flow cytometric analysis was applied to determine the alterations in cell cycle distributions in NSCLC cell lines following sequential administrations of gemcitabine and sorafenib for 72 h. The concentrations of gemcitabine and sorafenib were used at IC50 levels. Columns in the graphs depict cell cycle phase distribution in (A) A549 and (B) PC-9 cells following the administration of the exposure schedules indicated. G→S, S→G and S+G refer to gemcitabine followed by sorafenib, sorafenib followed by gemcitabine and concurrent administration, respectively. NSCLC, non-small cell lung cancer; IC50, concentration resulting in inhibition of 50% of maximal cell growth.

Flow cytometry was applied to evaluate the cell cycle phase distributions in EGFR-TKI-sensitive and EGFR-TKI-resistant cells following single-drug, sequential and concurrent administration of gemcitabine and sorafenib (Fig. 5). Following sorafenib treatment, the proportion of A549 and PC-9 cells in G0/G1 phase increased relative to control cells (P<0.05). Following treatment with gemcitabine alone, the fraction of A549 and PC-9 cells in S phase increased (P<0.05). Treatment with gemcitabine followed by sorafenib resulted in an increase in cells in the S and G2/M phases (P<0.05). By contrast, when cells were exposed to the reversed sequence, the proportion of cells in the S phase decreased (P<0.05).

### Gemcitabine-mediated activation of downstream signaling pathways

**Figure 6.**
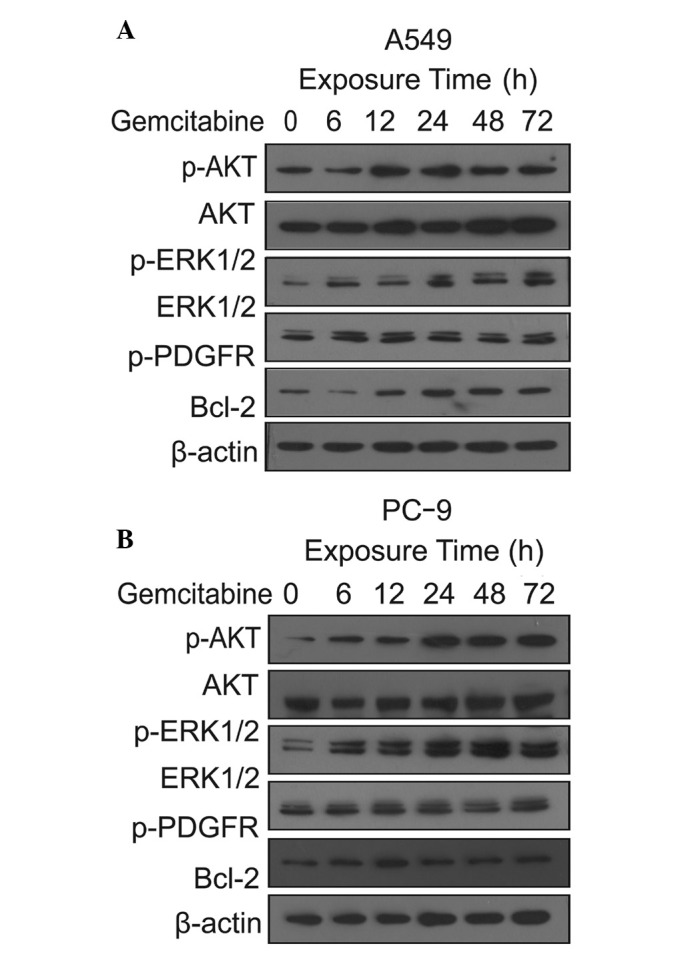
Downstream signaling pathways were activated by gemcitabine alone with increasing exposure time. Cells were exposed to concentrations of gemcitabine (three times IC50) for the indicated time; proteins of downstream signaling pathways were then analyzed by western blot analysis with corresponding antibodies. The expression levels of the proteins were detected in (A) A549 and (B) PC-9 cells. IC50, concentration resulting in inhibition of 50% of maximal cell growth; ERK, extracellular signal-regulated kinase; PDGFR, platelet-derived growth factor receptor.

To further evaluate the potential synergistic mechanisms of gemcitabine and sorafenib, the effects of gemcitabine on the downstream AKT and ERK signaling pathways and the anti-apoptotic Bcl-2 protein were detected by western blot analysis in PC-9 and A549 cells. The MEK/ERK and PI3K/AKT pathways are critical for proliferation and survival. In the two cell lines, we found that the level of p-AKT gradually increased from 0 to 24 h and lasted for 72 h when cells were exposed to gemcitabine at three times the IC50 concentration. Similarly, we observed an increase in p-ERK level in the two cell lines induced by gemcitabine alone. We also observed gemcitabine induced an increase in the Bcl-2 level in PC-9 and A549 cells. The effects of exposure to gemcitabine were not significantly different in EGFR-mutated and K-Ras-mutated cells (Fig. 6).

### Enhanced anti-proliferative effects of the schedule of gemcitabine followed by sorafenib

**Figure 7.**
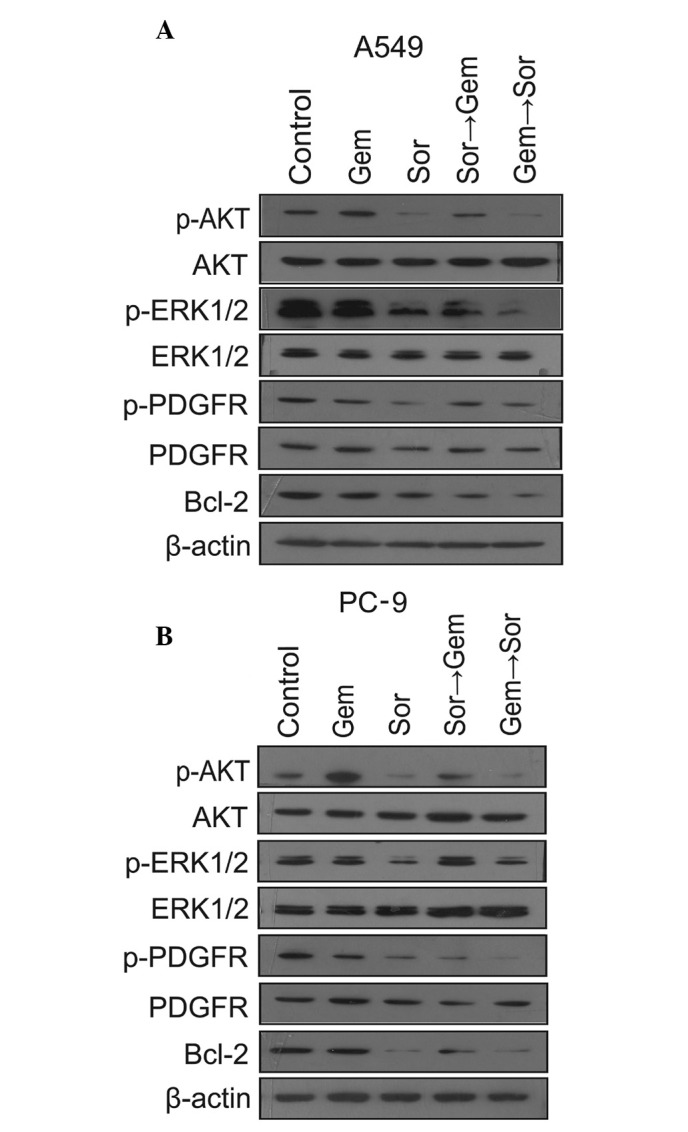
Effects of sorafenib and gemcitabine alone and in the sequential exposure schedules for 72 h on the expression levels of upstream and downstream signaling pathways in (A) A549 and (B) PC-9 cells were evaluated by western blot analysis. Gem (gemcitabine), Sor (sorafenib) and sequential exposures of Sor→Gem (sorafenib followed by gemcitabine) and Gem→Sor (gemcitabine followed by sorafenib) affected the expression levels of molecules in upstream and downstream signaling pathways. ERK, extracellular signal-regulated kinase; PDGFR, platelet-derived growth factor receptor.

The effects of sorafenib and gemcitabine as single agents and in different exposure schedules on cell signaling pathways in A549 and PC-9 cells were evaluated. As shown in Fig. 6, after 24 h exposure to gemcitabine at IC50 concentration, the levels of p-AKT were upregulated, but p-ERK did not change significantly, compared with Fig. 6. This result implies that gemcitabine increased the levels of p-ERK at a certain concentration and exposure time. We found that sorafenib downregulated the levels of p-PDGFRβ, p-AKT, p-ERK and Bcl-2 in A549 and PC-9 cells compared with unex-posed cells. We also found that the decreased Bcl-2 level in PC-9 cells was more significant than in the A549 cells. When the two cell lines were exposed to the sequence of gemcitabine followed by sorafenib, the levels of p-PDGFRβ, p-AKT, p-ERK and Bcl-2 were downregulated compared with sorafenib followed by gemcitabine. Upregulation of p-PDGFRβ, p-AKT, p-ERK and Bcl-2 expression levels was observed following the exposure sequence of sorafenib followed by gemcitabine compared with the reversed sequence (Fig. 7). However, compared with the control cells, there was no significant variation in the total PDGFR, ERK and AKT expression.

## Discussion

Platinum-based chemotherapy has become the mainstay treatment for advanced NSCLC (35). Although traditional cytotoxic chemotherapy improves patient survival, treatment options remain limited for patients with advanced NSCLC. Recently, targeted anticancer drugs, including EGFR-TKIs, have been approved for the treatment of lung cancer (36). However, EGFR-TKIs, such as gefitinib and erlotinib, have no effect in the majority of K-Ras mutation NSCLC tumors (37). The development of new treatment strategies for NSCLC patients is thus an important clinical goal.

There is multilevel cross-stimulation among the targets in lung cancer. When only one pathway is blocked, others act as salvage or escape mechanisms for cancer cells. Anticancer agents that interfere at different stages and avoid escape or salvage mechanisms may be more effective than single targeted agents (38). Therefore, multi-targeted TKIs that block multiple signaling pathways are considered to be more effective therapeutic agents for cancer. Sorafenib is a novel, multi-kinase inhibitor that targets tumor proliferation and tumor angiogenesis (20). It has been approved for the treatment of advanced renal cell cancer and is currently being evaluated for the treatment of other tumors (20).

The present study was performed in EGFR-TKI-sensitive PC-9 (EGFR mutant/wild-type K-Ras) and EGFR-TKI-resistant A549 (wild-type EGFR/mutant K-Ras) human lung cancer cell lines to investigate the anti-proliferative effects of sorafinib as a single agent and in different schedules in combination with gemcitabine. We found that sorafenib and gemcitabine exhibited dose-dependent growth inhibition of cell growth as single agent treatment in PC-9 and A549 lung cancer cells. This result suggests that sorafenib is efficacious for growth inhibition in EGFR-mutated and K-Ras-mutated NSCLC cell lines. The strongest synergism was observed upon administration of gemcitabine followed by sorafenib in the two cell lines, whereas antagonistic interactions were observed upon administration of sorafenib followed by gemcitabine. Similar to our observations, a previous *in vivo* study demonstrated that the administration of gemcitabine followed by sorafenib had synergistic effects in NSCLC cells (39). Therefore, this sequential administration of sorafenib and gemcitabine may benefit patients with K-Ras mutations.

In our study, A549 and PC-9 cells were arrested by sorafenib at G1 phase, while gemcitabine caused cells to accumulate in S phase. The synergism and antagonism effect may be explained by these effects between the two drugs. A549 and PC-9 cells were first arrested at the G1 phase by sorafenib, thereby the proportion of cells in S phase decreased, resulting in weakened cell cycle-specific cytotoxicity of gemcitabine. In the reverse sequence, gemcitabine arrested cells in the S phase, inhibiting cell mitosis and multiplication, then the subsequent sorafenib suppressed the cell growth, thereby increasing the proportion of apoptotic cells. Similar to our observation, previous studies concerning antagonism between sorafenib and chemotherapy have reported that cells were arrested firstly by sorafenib at G1 phase, interfering with the cytotoxicity effects in S phase of the cell cycle-specific drugs (40,41).

The difference in the sequence-dependent anti-proliferative effects of sorafenib and gemcitabine may also result from growth signaling pathways. We found that gemcitabine enhanced the expression levels of molecules in downstream signaling pathways, for example, increasing the levels of p-AKT and p-ERK in A549 and PC-9 cells. Similar to our results, a previous study reported that cell signaling pathways may be gradually activated by chemotherapy (42). p-ERK and AKT play important roles in tumor cell proliferation, but gemcitabine induced ERK and AKT phosphorylation, leading to the prevention of apoptosis.

We have shown that sorafenib inhibited the activity of the upstream receptor PDGFRβ and decreased the levels of the downstream p-AKT, p-ERK and Bcl-2 in the A549 and PC-9 cell lines after 72 h exposure. Sorafenib inhibited the PDGFRβ-dependent activation of the PI3K/AKT and MAPK pathways, thereby decreasing the levels of p-AKT and p-ERK (43). Sorafenib decreased the activity of C-RAF and B-RAF in the RAF/MEK/ERK signaling pathway, meaning that the level of p-ERK may also be downregulated directly. However, a previous study has reported that sorafenib failed to inhibit p-ERK in NSCLC cell lines with K-Ras mutations (28). The conflicting results may be attributed to a shorter exposure to sorafenib. It means that prolonging the exposure time of sorafenib may decreased the level of p-ERK.

Sorafenib inhibited the expression of Bcl-2 in the two cell lines, mainly by the simultaneous inhibition of the ERK and AKT downstream pathways. We also found that the decreased Bcl-2 level in PC-9 cells was more significant than in the A549 cells. We conclude that sorafenib has stronger cytotoxicity effects in lung cancer cells with K-Ras mutation.

We also found that the expression of p-ERK and p-AKT differed in response to each combinatorial treatment. When gemcitabine was administered first, a significant decrease in p-AKT and p-ERK levels was observed in the A549 and PC-9 cell lines. Conversely, when sorafenib was administered first, the levels of p-AKT and p-ERK were decreased and then upregulated by subsequent exposure to gemcitabine. The sorafenib-induced decrease in p-ERK and p-AKT appears to be reversible. These observations of p-AKT and p-ERK in NSCLC cells may explain the synergistic and antagonist growth inhibitory effects observed in A549 and PC-9 cells treated with sorafenib and gemcitabine.

In conclusion, we found that sorafenib exhibited significant growth inhibition in EGFR-TKI-sensitive and EGFR-TKI-resistant NSCLC cells. Moreover, regardless of the mutation status of EGFR and K-Ras, the sequential administration of gemcitabine followed by sorafenib was an optimum schedule against NSCLC. These data encourage the development of sorafenib as a single targeted therapy or in combination with cytotoxic chemotherapy drugs for treatment of NSCLC. Our study used an *in vitro* model and was unable to test the antiangiogenic effects of sorafenib, so further *in vivo* studies are required to explore sorafenib as a single agent and the schedule-dependent administration of sorafenib plus gemcitabine in NSCLC.

## Figures and Tables

**Table I.** IC50 values of sorafenib and gemcitabine were determined by MTT.

IC50	A549	PC-9
Gemcitabine	10.38±0.80 nM	8.38±0.64 nM
Sorafenib	5.91±0.22 *μ*M	6.13±0.14 *μ*M

IC50, concentration resulting in inhibition of 50% of the maximal cell growth.
